# Si Nanocrystals/ZnO Nanowires Hybrid Structures as Immobilized Photocatalysts for Photodegradation

**DOI:** 10.3390/nano10030491

**Published:** 2020-03-09

**Authors:** Yaozhong Zhang, Rajib Mandal, Daniel C. Ratchford, Rebecca Anthony, Junghoon Yeom

**Affiliations:** 1 Department of Electrical and Computer Engineering, Michigan State University, East Lansing, MI 48823, USA; yzzhang@egr.msu.edu; 2 Department of Mechanical Engineering, Michigan State University, East Lansing, MI 48824, USA; rajib.mandal@intel.com (R.M.); ranthony@msu.edu (R.A.); 3 Code 6178, Chemistry Division, U.S. Naval Research Laboratory, Washington, DC 20375, USA; daniel.ratchford@nrl.navy.mil

**Keywords:** zinc oxide nanowires, silicon nanocrystals, hybrid nanostructures, photodegradation, photoluminescence

## Abstract

Numerous semiconductor-based hybrid nanostructures have been studied for improved photodegradation performance resulting from their broadband optical response and enhanced charge separation/transport characteristics. However, these hybrid structures often involve elements that are rare or toxic. Here, we present the synthesis and material characterization of hybrid nanostructures consisting of zinc oxide (ZnO) nanowires (NWs) and silicon nanocrystals (Si-NCs), both abundant and environmentally benign, and evaluate them for photodegradation performance under various illumination conditions. When incorporating Si-NCs into the vertically-aligned ZnO NWs immobilized on substrates, the resulting photocatalysts exhibited a narrowed band gap, i.e., more responsive to visible light, and enhanced charge separation at the interface, i.e., more reactive species produced for degradation. Consequently, the hybrid Si-NCs/ZnO-NWs displayed a superior photodegradability for methylene blue under UV and white light in comparison to the pristine ZnO NWs. Based on the optical measurements, we hypothesize the band structures of Si-NCs/ZnO-NWs and the potential mechanism for the improved photodegradability.

## 1. Introduction

Various forms of ZnO nanostructures have attracted considerable attention as one of the most efficient catalysts for photocatalytic degradation, due to their high specific surface-area, direct bandgap, rapid carrier transport, and strong oxidation ability [1,2]. ZnO is also an abundant, environmentally friendly material, and it is easy and inexpensive to grow in nanostructures [3,4]. Under UV light irradiation, organic pollutants in waste water streams can be decontaminated in the presence of ZnO nanostructures, where the photogenerated electrons and holes travel to the catalyst surface and produce highly oxidizing species such as hydroxyl radicals and super oxide ions responsible for degradation reactions [5]. Despite their remarkable photocatalytic activity, ZnO nanostructures suffer from the rapid recombination of photogenerated electron–hole pairs and low utilization of the solar spectrum due to their limited spectral response to visible light [5]. Since the UV region covers a small fraction (4%~5%) of the solar spectrum, photocatalysts with better optical absorption in the visible spectrum (~43% of solar spectrum) lead to the increased utilization of solar energy. The key to an improved photodegradation performance under sunlight is the development of functional materials that extend the absorbance spectrum to the visible region, and enhance photoexcited charge separation for a reduced electron–hole recombination.

Tremendous efforts have been made to boost the optical response of ZnO in the visible region, including metal doping (e.g., Ag, Au, Cu) [2,5], non-metal doping (e.g., N, C, S) [2,6], and coupling with narrow-bandgap semiconductors [7]. The latter two approaches to hybrid photocatalytic materials are especially promising because ZnO-metal or ZnO-semiconductor heterojunctions suppress carrier recombination by separating electrons and holes at the interface, and enhance the optical absorption by effectively narrowing down the bandgap [7]. However, noble metal nanoparticles are relatively expensive, and the metal loading and deposition method needs to be carefully controlled for optimal performance (this is especially important for a ZnO core decorated with metal nanoparticles). Instead, hybrid materials of ZnO and semiconductor nanostructures with a narrow bandgap exhibit superior charge separation, as well as enhanced light absorption capability, since both base and add-on materials can directly absorb light energy [7]. To date, ZnO has been coupled with various narrow-bandgap semiconductors for better photocatalytic functionality, including In_2_S_3_ [8], Fe_3_O_4_ [9], and CuWO_4_ [7]. Here, a relatively unexplored element that forms a hybrid structure with ZnO will be investigated in the context of photocatalysis—ZnO/Si heterogeneous nanostructures. Si is highly advantageous because of its abundance and environmental friendliness, and its nanostructures are well-studied in terms of fabrication and characterization. In particular, Si nanocrystals (Si-NCs) have been extensively investigated as a non-toxic, chemically stable, and bandgap-tunable component for solar cells [10], light emitting devices [11,12], and other optoelectronic applications [13]. Therefore, Si-NCs are a promising material to form ZnO-based heterostructures for environmentally-friendly and visible-light active photocatalysts.

Recyclability is another important factor in developing photocatalysts for water decontamination [14]. Most photocatalysts are in the form of spherical nanoparticles, and are synthesized and tested in a suspension, requiring an additional process to remove the photocatalysts from the treated water. Immobilized photocatalysts could eliminate this costly separation step [14], but their performances are limited due to the reduced surface area of the photocatalysts that are partially embedded into (or deposited onto) the substrate. One approach to increasing the effective surface area of immobilized photocatalysts is to harness vertically grown, one-dimensional nanostructures (e.g., nanowires/nanorods [15] and nanotubes [16]) for improved photocatalytic performance. In particular, ZnO nanowires (ZnO NWs) are easier to directly grow on a substrate at a high aspect-ratio compared to other semiconducting photocatalysts (e.g., TiO_2_), and exhibit high crystallinity for efficient charge transport. There are several reports on ZnO-NW-based heterostructures for photocatalytic applications [17] but, to best of our knowledge, Si-NCs in conjunction with ZnO NWs have not yet been investigated for photodegradation and other optoelectronic applications.

In this work, the hybrid structures of Si nanocrystals/ZnO nanowires (Si-NCs/ZnO-NWs) were synthesized as immobilized photocatalysts for pollutant degradation. While the enhanced photodegradability of the hybrid structure was demonstrated under visible light in our previous work [3], the detailed accounts of the hybrid structures’ optical responses and band structures were missing in [3], and thus are the subject of this study. The Si-NCs, which possess a tunable bandgap (1.2 ~2.8 eV) [18], extend the light absorption of ZnO NWs into the visible spectrum and facilitate the charge carrier separation. The morphology of the hybrid nanostructures significantly influenced the photocatalytic performance, and therefore the systematic study of the Si-NC loading effect on the light absorption, photoluminescence, and photodegradation was conducted under various illumination conditions, to reveal the possible mechanism of the enhanced photodegradation from the Si-NCs/ZnO-NW hybrid structures and the optimal Si-NC loading.

## 2. Materials and Methods

### 2.1. Synthesis of ZnO NWs

All reagents used in this experiment were commercially available without further purification. ZnO NWs were synthesized via the hydrothermal method [3]. The detailed process is given as follows: a ZnO seed solution was prepared by adding 10 mM Zn(CH_3_COO)_2_·2H_2_O (99.9% Sigma Aldrich Corp., St. Louis, MO, USA) into ethanol (200 prove, Fisher Scientific Inc., Hampton, NH, USA) and stirred at 58 °C for 2 h. The mixed solution was spin-coated onto a cleaned glass or Si substrate (25 by 25 mm in size) multiple times at room temperature. The substrate was then kept at 150 °C for 1 h to promote the adhesion of the seed particles to the substrate. The growth solution was composed of 25 mM Zn(NO_3_)_2_·6H_2_O (Sigma Aldrich) and 25 mM hexamethylenetetramine (HMTA, Sigma Aldrich). Prior to the synthesis, the growth solution was preheated in a convection oven at 90 °C for 1 h to achieve a thermal equilibrium. The substrate was then immersed into the solution at 90 °C for 6 h for ZnO NW growth. After removal from the solution, the substrate with ZnO NWs was thoroughly rinsed by DI water and blown dry. A 3-h-long dehydration step at 60 °C was carried out for the substrate afterwards.

### 2.2. Synthesis of Si Nanocrystals

Silicon nanocrystals (Si-NCs) were synthesized using a non-thermal plasma reactor in a fully gas-phase process. This process was originally developed by Mangolini et al. [19] and has been well-studied for several other nanoparticles’ synthesis [20,21]. Besides producing high-quality nanoparticles, gas phase synthesis has several other advantages, including accurate size control, adjustable particle structure, lesser degree of agglomeration, and hydride-terminated surfaces. The flow-through plasma reactor consists of a quartz tube with varying outer diameters of 0.5 and one inch in the top and bottom parts, respectively. The lengths of the contraction and expansion region are seven and five inches, with a total length of one foot. Argon (Ar, Airgas Inc., Radnor, PA, USA) was used as a background gas, with silane (SiH4, 1% in Ar, Praxair Inc., Danbury, CT, USA) as the precursor for the Si-NCs. The gas mixture was flown through the quartz tube, around which a pair of copper electrodes were wrapped. Hydrogen (H_2_, Airgas Inc.) was flowed through the sidearm into the effluent of the plasma, to provide additional hydrogen termination and reduce dangling bond densities [22]. The gas flow rates were 30 standard cubic centimeters per minute (sccm) of Ar, 80 sccm of Ar/SiH_4_, and 50 sccm of H_2_, and the pressure in the reactor was kept constant at 2.75 Torr using a slit-shaped orifice. Radiofrequency (rf) power at 25 W was applied to the copper electrodes, using a 13.56 MHz power supply and an impedance-matching network. The Si-NCs synthesized using this recipe were approximately 4–5 nm in diameter.

### 2.3. Surface Functionalization Process for Si-NCs

As-produced Si-NCs were surface-functionalized in a thermal hydrosilylation reactor [23]. Si-NCs were collected via diffusion onto a stainless steel mesh. A functionalization solution was prepared by mixing mesitylene (Sigma Aldrich) and 1-decene (Sigma Aldrich) in a volume ratio of 5:1, and was dried and degassed using molecular sieves (size 4 Ǻ) and nitrogen bubbling. This solution was then mixed with Si-NCs (concentration approximately 1 mg/mL) and sonicated for a few minutes (until the Si-NCs were no longer agglomerated at the bottom of the container). This solution was heated in a refluxer for about 2 h under a nitrogen flow at 170 °C, to recondense any evaporated solvents. When the reaction was complete, the solution was a clear colloidal liquid rather than the original cloudy dispersion. The surface-passivated Si NCs were then dried and redispersed in toluene (Sigma Aldrich). The quantum yield (QY) immediately after the functionalization was 40%–60%.

### 2.4. Preparation of Hybrid Structure

After the surface functionalization, the Si-NCs of 5 mg/mL were spin-coated on the as-prepared ZnO-NW substrate at 500 rpm for various times to control the Si-NC loading. In this experiment, three different loadings of Si NCs, i.e., 5, 10 and 15 µL, were applied to the ZnO-NW substrates and named as ZS1, ZS2 and ZS3, respectively. For the comparison, a pristine ZnO-NW substrate was prepared and denoted as ZS0. The subsequent dehydration step was performed for these hybrid structures at 40 °C in vacuum for 3 h to improve the adhesion of Si-NCs to ZnO NWs.

### 2.5. Materials Characterization

The morphology, crystallinity and composition of three Si-NCs/ZnO-NWs hybrid structures and the pristine ZnO NW sample were characterized using micro-Raman spectroscopy (Alpha300A, Witec, Ulm, Germany), X-ray diffraction (XRD, Bruker-AXS, Billerica, MA, USA), scanning electron microscopy (SEM, Hitachi S-4700II, Tokyo, Japan), energy-dispersive X-ray spectrometer (EDS, JEOL 6610LV, Tokyo, Japan), and transmission electron microscopy (TEM, JEOL 2200FS), and Fourier transform infrared spectroscopy (FTIR, Bruker Alpha, Billerica, MA, USA). The optical response of the as-prepared samples was obtained by photoluminescence (PL) spectroscopy, with the excitation wavelength at 337 nm and the emission filtered by a 345 nm long pass filter. The PL lifetime of the Si NCs was collected using a pulsed nitrogen laser excitation source (λ = 337 nm, pulse duration = 10 ns, and repetition rate = 20 Hz) and a photomultiplier tube for detection. The signal was filtered by a 345 nm long pass filter and a 675 ± 50 nm band pass filter before detection to spectrally isolate the emission from the Si NCs. Diffuse reflectance from the samples was measured with a PerkinElmer LAMBDA 1050 UV/Vis/NIR Spectrophotometer (Waltham, MA, USA) with an integrating sphere attachment.

### 2.6. Photodegradation Assessment

Under UV light: The photocatalytic degradation was carried out in an aluminum-foil-sealed case equipped with a magnetic stir plate. Two 9 W fluorescent tubes (λ = 375 nm, Philips, NJ, USA) were positioned 13 cm above a dye solution container. Methylene blue (MB, 1.5%, Sigma-Aldrich) was diluted to 10 mM and used as a model pollutant. The substrate (cut into 5 by 5 mm) with the Si-NC/ZnO-NW hybrid structures was immersed in the dye solution (12 mL). Before irradiation, the dye solution was kept in the dark for 30 min to establish the absorption–desorption equilibrium. Upon irradiation, 1 mL of solution was extracted from the container at a constant time interval (typically 30 min) and centrifuged at 10,000 rpm for 2 min. The purified solution was then examined by a UV-vis spectrometer (Perkin Elmer LAMBDA 900) to measure the absorption of the remaining methylene blue. The experiment was repeated three times to determine the relative error compared to that of a substrate with ZnO NWs.

Under visible light and white light: A 250 W Halogen light (spectral range 265–669 nm) was employed as a white light source. Before irradiation, a solar-filtered transparent film (ENP® ultra clear 7225) was placed between the light source and the dye solution. This film rejects 96% and 75% of the UV and infrared light, respectively, and transmits 72% of the visible light. Except for the 30-cm-distance of light source, the remaining procedures were the same as those of the photodegradation experiment under UV light. The temperatures 6 h after the light was turned on were 30 °C (dye solution side) and 50 °C (light source side) on both sides of the transparent film. The experiments were repeated under the same condition in the absence of solar-filtered transparent film for the white light irradiation. The solution temperatures in all experiments were kept below 28 °C during 6-h irradiation.

## 3. Results and Discussions

### 3.1. Materials Characterization

**Figure 1:**
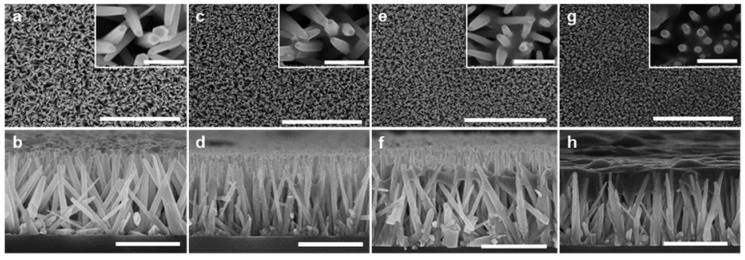
Top-down and cross-sectional SEM images of (**a**,**b**) the pristine ZnO-NW sample—ZS0; the ZnO-NWs/Si-NCs hybrid samples with the three different Si-NC loadings, (**c**,**d**)—ZS1, (**e**,**f**)—ZS2, and (**g**,**h**)—ZS3. Scale bar: (**a,c,e,g**) 10 µm in large view and 500 nm in the inset, (b,d,f,**h**) 2 µm.

The ZnO NWs were synthesized on a glass or silicon substrate by the standard hydrothermal method, and the resulting NW structures were consistent with those reported in the literature [24]. The Si NCs were synthesized via a non-thermal low-pressure plasma process, and subsequently functionalized to stabilize the nanocrystals and reduce the surface oxidation. The Si NC diameter was carefully controlled to approximately 5 nm. Figure 1 shows the top-down and cross-sectional scanning electron microscopy (SEM) images of the pristine ZnO-NW sample (ZS0) and the ZnO-NWs/Si-NCs hybrid samples with the three different Si-NC loadings (ZS1, ZS2, and ZS3). The vertical ZnO NWs had a hexagonal shape, with a diameter of 50 to 80 nm and a length of 1.5 to 2 μm. They were uniformly grown on the Si or glass substrate, and the average density of the ZnO NWs was roughly 10/µm^2^.The Si-NC loading was adjusted by the number of times that spin-coating of a given aliquot (5 μL) was applied to the ZnO-NW substrates.

During the spin-coating process, the Si-NC suspension partially embedded into the voids between the ZnO NWs, and deposited the NCs on the NW surface. As seen from Figure 1a,c,e,g, increased loading gradually filled the gaps between the ZnO NWs with Si-NCs. The cross-sectional views (Figure 1b,d,f,h) reveal that most Si-NCs agglomerated around the NW tips after the deposition and tented over the ZnO NWs. The sample with the highest Si-NC loading (ZS3) shows the formation of the continuous layer (dark cloudy layer in Figure 1h). If the loading was further increased, the extra Si-NCs fully encapsulated the entire NW array and created a thick overlayer (see Supplementary Information Figure S1). When the hybrid structure with heavy Si-NC loading was irradiated, most of the UV and visible photons were absorbed by the Si-NCs, and did not reach the ZnO NWs. Therefore, it is difficult to consider it as the intended hybrid structure, because the optical response is dominated by the thick Si-NCs. There should be an appropriate Si-NC thickness (or coverage) which avoids significant blockage of the light transmitting to the ZnO NWs and exploits the benefits of the hybrids.

Elemental analysis with energy-dispersive x-ray spectroscopy (EDS) performed on these four samples (ZS0, ZS1, ZS2, and ZS3) revealed the relative mass ratio of Zn and Si. Figure S2 in the Supplementary Information shows the top-down SEM views of the four samples and corresponding EDS elemental mapping spectra for Zn, O, and Si. The elemental ratios of Zn to Si for ZS1, ZS2 and ZS3 are 47.12, 12.63 and 7.2, respectively, demonstrating the increased Si for higher Si NC loading. The Si was evenly distributed across the images for all the samples, indicating that Si-NCs were applied homogenously. The uniform Zn and Si distribution in ZS1, ZS2 and ZS3 confirms the intimate attachment of Si NCs to ZnO NWs over the substrate, where the elemental concentration of Si fluctuates along the length of the ZnO NW array.

**Figure 2:**
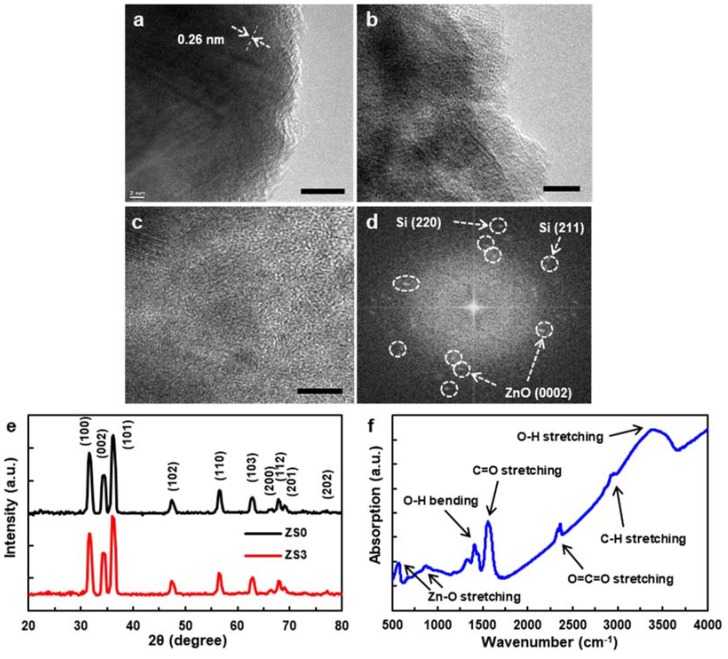
TEM images of (**a**) the pristine ZnO NW (ZS0) and (**b**–**d**) the Si-NCs/ZnO-NWs hybrid nanostructure (ZS2), (**e**) XRD patterns of ZS0 and ZS3, (**f**) FTIR Spectra of ZS0. Scale bar: (**a**–**c**) 10 nm.

Figure 2a shows the TEM image of the pristine ZnO NW (ZS0), in which the lattice fringes (~0.26 nm) are in good agreement with the distance between two (0001) planes of wurtzite ZnO [25]. The TEM image of one of the Si-NCs/ZnO-NWs hybrid nanostructures (ZS2) is shown in Figure 2b,c. The Si NCs of various crystal orientations continuously surrounded the ZnO NWs. The lattice fringes indicate that both the ZnO NWs and Si NCs maintained their crystalline structures. The XRD patterns of ZS0 and ZS3 are shown in Figure 2e. The peaks of both samples corresponded to the wurtzite phase of ZnO NWs [24,26], and the peaks with a higher intensity depict the preferential growth direction [0001] of the ZnO NWs. The absence of Si-specific peaks from ZS3 (red) presumably reflects the low volume fraction of Si NCs. The Fourier Transform Infrared Spectra (FTIR) of ZS0 was collected in the range of 500 ~ 4000 cm^−1^. As displayed in Figure 2f, the peaks at 3393 and 1413 cm^−1^ correspond to the stretching vibration mode and bending vibration of the O-H group, respectively. A strong peak at 1557 cm^−1^ is attributed to the C=O stretching vibration. The peaks observed at 886 and 570 cm^−1^ indicate Zn-O stretching, while the peaks at 2361 and 2966 cm^−1^ can be assigned to O=C=O and C-H stretching, respectively [27,28].

**Figure 3:**
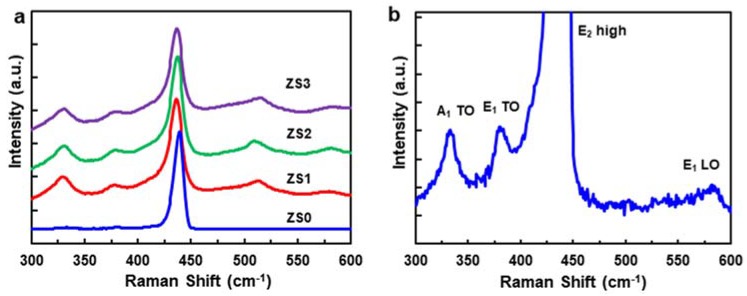
(**a**) Room-temperature Raman scattering spectra of the pristine ZnO NWs (ZS0) and three Si-NCs/ZnO-NWs hybrid structures (ZS1, ZS2, and ZS3) and (**b**) the zoom-in Raman scattering spectra of ZS0.

Figure 3a shows the room-temperature Raman scattering spectra of the pristine ZnO NWs (ZS0) and three Si-NCs/ZnO-NWs hybrid structures (ZS1, ZS2, and ZS3), and confirms their crystallinity. For ZS0, four Raman peaks are shown in Figure 3b, where the peak with the highest intensity at 440 cm^−1^ is the E2 (high) mode [29]. The three other peaks, located at 340, 378, and 584 cm^−1^, are assigned to the A1 (TO), E1 (TO), and E1 (LO) modes, respectively. Another peak at 101 cm^−1^ (data not shown) for the E2 (low) mode matches the previous reports [30]. The Raman spectra for the hybrid structures show an additional peak at 515 cm^−1^, corresponding to the TO of Si NCs. The peak position of the Si NCs detected at 515 cm^−1^ differs from that of single-crystal Si at 521 cm^−1^, which we attribute to the lattice expansion of Si NCs [31]. Furthermore, the peak with an asymmetric shape deviates from the Lorentzian function, indicating that the Si NCs are sub-micron in scale, consistent with electromagnetic radiation theory [32,33,34]. The Raman peaks of the ZnO NW for different Si-NC loadings, especially the A1 phonons, show no significant peak shift or broadening, suggesting that the hybrid structures are stably and reproducibly synthesized.

### 3.2. Photoluminescence Measurements

**Figure 4:**
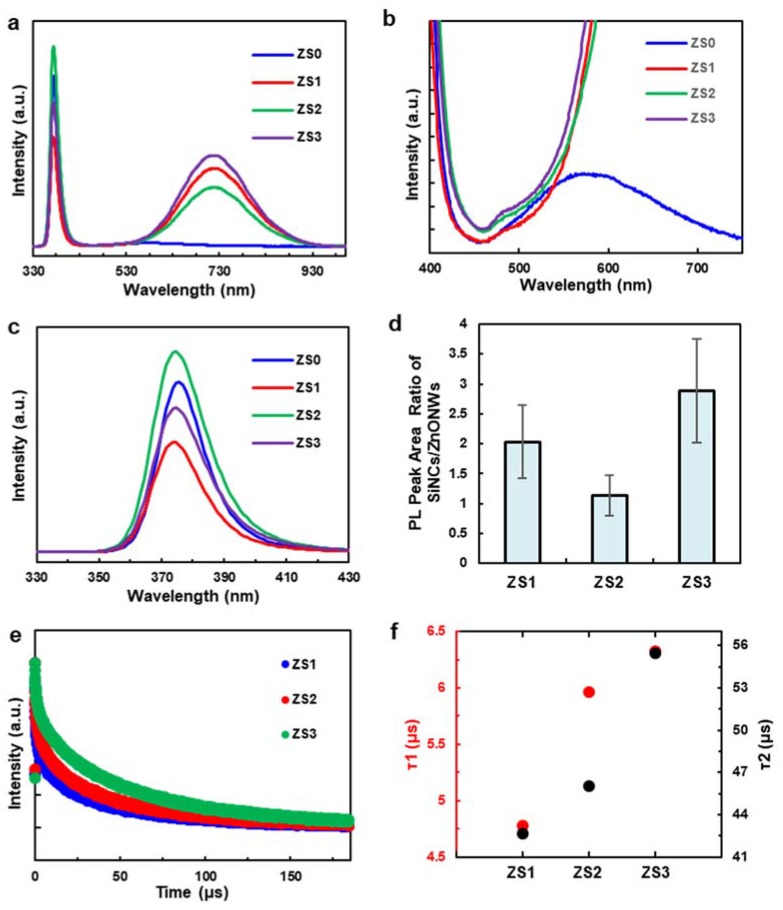
(**a**) Room temperature PL spectra of the pristine ZnO NWs (ZS0) and three Si-NCs/ZnO-NWs hybrid structures (ZS1, ZS2, and ZS3), (**b**–**c**) the zoom-in PL spectra of ZS0, ZS1, ZS2 and ZS3, (**d**) the NBE-normalized factors of ZS0, ZS1, ZS2 and ZS3, (**e**) the time-resolved PL decay of the Si NCs on the ZnO NWs, (**f**) the fitted fast and slow PL decay times of ZS1, ZS2 and ZS3.

Room temperature photoluminescence (PL) spectra (see Figure 4a) were measured to investigate how Si-NCs affect the luminescent properties of the hybrid structures. The narrow peaks at 380 nm from all four samples can be attributed to the near-band-edge (NBE) emission of ZnO, which is mostly related to the recombination of free excitons through an exciton–exciton collision process [35]. Another small, broad peak at 570 nm from ZS0 (see Figure 4b) may originate from the recombination of electrons with oxygen vacancies and photogenerated holes in the valence band, which is typically related to the defect concentration of ZnO [30]. Therefore, the strong NBE emission and weak green emission in the PL spectrum suggest that the hexagonal-shaped ZnO NWs have good crystallinity and optical properties [36].

The magnified plot of Figure 4b shows that the weak, defect-related ZnO emission peaks for ZS1, ZS2 and ZS3 are mostly overwhelmed by the strong and broad luminescence peaks of Si-NCs in the range between 500 and 900 nm [37]. The ZnO NBE emission peak was suppressed at the lowest Si-NC loading (ZS1), increased at a higher loading (ZS2), then decreased again at an even higher loading (ZS3) (see the zoom-in plot in Figure 4c). To account for this irregular trend, the NBE-normalized factors (defined as the PL peak area ratio of Si-NCs to ZnO NWs) of ZS1, ZS2 and ZS3 are plotted (the factor of ZS0 is close to 0). The emission ratio in Figure 4d displays a different trend than the Si-NCs loading protocol (i.e., the linear increment of quantity), which has an increment from ZS0 to ZS1 and is followed by a reduction at ZS2. We hypothesize that this trend is linked to charge recombination and surface passivation on the ZnO NWs. From ZS0 to ZS1, when incorporated with a small quantity of Si NCs, the ZnO NWs were partially covered by the NCs (see Figure 1b,f). In this case, both ZnO NWs and Si NCs are instantly activated under light irradiation, while the Si/ZnO junction facilitates the separation of photogenerated charge carriers. Meanwhile, the surface passivation of the ZnO NWs derived from the Si NCs reduces the surface defect sites (i.e., recombination centers), thus lowering radiative recombination [38]. According to the Si-NC loading protocol, ZS2 contained many more Si-NCs (see Figure 1c,g), and probably exceeded the number required for surface passivation. It has been suggested that charge carrier transport in ZnO/TiO_2_ hybrid nanostructures bends the ZnO conduction band (CB) upwards, due to electron trapping at the interface, which acts as an energetic barrier and impedes further electron transfer from the TiO_2_ [39]. Likewise, since most of the photons were absorbed by the Si-NCs, the excess electrons generated within the Si-NCs may have accumulated at the Si-NCs/ZnO-NWs interface and formed a barrier that hindered the charge carrier transport across the Si/ZnO junction. Consequently, radiative recombination within ZnO NWs was promoted, leading to a reduction in the NBE-normalized factor. For ZS3, the tips of the ZnO NWs were completely covered by the Si-NC layer (see Figure 1d,h), which blocked the photons from reaching the ZnO NWs and creating excitons. This “screening” effect undercuts the gains obtained from the enhanced charge transfer at the hybrid interfaces, and thus results the increment of the factor. Only the Si-NCs in direct contact with, or close proximity to, the ZnO NWs passivate the surface and inject charge.

Figure 4e shows the time-resolved PL decay of the Si NCs on the ZnO NWs (signal filtered at 675 ± 50 nm). Each PL decay is normalized by its maximum intensity for ease of comparison. The corresponding fast and slow decays of the three hybrid nanostructures were identified through a bi-exponential fit (R^2^ > 0.99 for all fits) using $y=y_{0}+A_{1}\exp\left(-t/\tau_{1}\right)+A_{2}\exp\left(-t/\tau_{2}\right)$. According to Figure 4f, the fitted fast PL decay time constants (τ_1_) are 4.78, 5.96 and 6.32 μs for ZS1, ZS2, and ZS3, respectively, while the corresponding slow PL decay time constants (τ_2_) are 42.64, 46.06 and 55.45 μs. Previous studies from the literature correlated the fast PL decay in the Si NCs to the average carrier lifetime of a non-radiative recombination process, while the slow one describes radiative recombination [40,41]. The latter mechanism is more relevant to NBE emission, so we emphasized τ_2_. The average exciton lifetime of the room-temperature PL process in pure ZnO NWs is reported to range from hundreds of picoseconds to a few nanoseconds [42,43], while the average exciton lifetime of Si NCs is approximately 80 μs [44]. In the current system, the shorter τ_2_ lifetime at lower Si-NC loading suggests that photogenerated electrons in the Si NCs recombine more quickly after being injected into the ZnO.

This reduction in PL lifetime for the Si-NCs may result from electron transport from the NCs into the ZnO NWs, as observed for quantum-dot-sensitized solar cells (QDSSCs) [45,46] and other hybrid NC/receptor materials based on cadmium chalcogenide nanocrystals. At high Si-NC loading, the PL lifetime is dominated by the long lifetimes that are typical of silicon nanocrystals. At lower Si-NC loading, the PL lifetime is reduced as a result of exciton dissociation and electron transport into the ZnO NWs, which is driven by the favourable energy band alignment between the two materials. Therefore, the exciton lifetime of the hybrid nanostructure is statistically shorter than that of the pure Si NCs, and the lifetime reduction is a function of the percentage of Si NCs involved in the charge transfer from Si to ZnO at their interface. In other words, ZS1 has the lowest τ_2_ because the majority of Si NCs in ZS1 interface with ZnO NWs, accelerating exciton recombination. τ_2_ steadily recovered to the rates of the pure Si NCs at a heavier Si-NC loading. Since the quantum yield of the Si NCs is approximately 40%, the radiative energy transfer lifetime of ZS1, ZS2 and ZS3 can be estimated to be 11.95, 14.9 and 15.8 μs, respectively [10].

### 3.3. Diffuse UV-Vis Spectra

**Figure 5:**
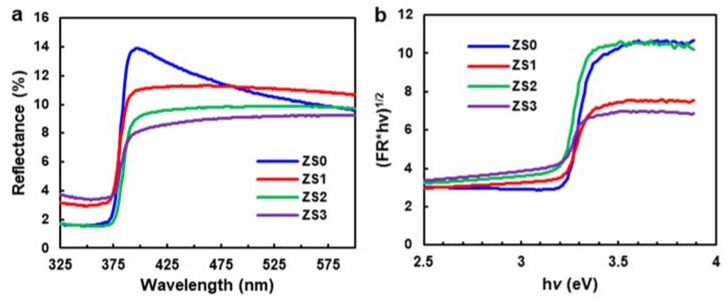
(**a**) Diffuse reflectance spectra of the pristine ZnO NWs (ZS0) and the Si-NCs/ZnO-NWs hybrid structures (ZS1, ZS2, ZS3), (**b**) the plot of the modified Kubelka–Munk function vs. the energy of exciting light.

Figure 5a displays the diffuse reflectance spectra of pristine ZnO NWs and Si-NCs/ZnO-NWs hybrid structures. In the UV-vis region, the diffuse reflectance of ZS0 is greater than that of the hybrid structures (in fact, ZS0 > ZS1 > ZS2 > ZS3), indicating stronger absorption for a higher Si-NC loading. The plot of the modified Kubelka–Munk function, (*F*(*R*)*hν*)^1/2^, vs. the energy of exciting light (*hν*), can be employed to calculate the optical band gap before and after the SiNC decoration [47,48] (see Figure 5b). Here, *F*(*R*) = (1 − *R*)^2^/2*R*, where *R* is the reflectance, *h* is the Planck constant, and *ν* is the frequency of light. The x-intercept values from the extrapolation of the linear slope to the photon energy in Figure 5b can be related to band gap energy. The calculated band gap energies of ZS0, ZS1, ZS2, and ZS3 are 3.22, 3.10, 3.14 and 3.07 eV, respectively. The maximum bandgap narrowing of 0.15 eV was observed upon the formation of ZnO/Si hybrid structures, which is similar to the other ZnO-semiconductor-based hybrid structures [48]. In this paper, the narrowed band gap of the hybrid structures is expected to extend the spectrum response of the catalyst and enhance photodegradation under visible light.

### 3.4. Photodegradation

**Figure 6:**
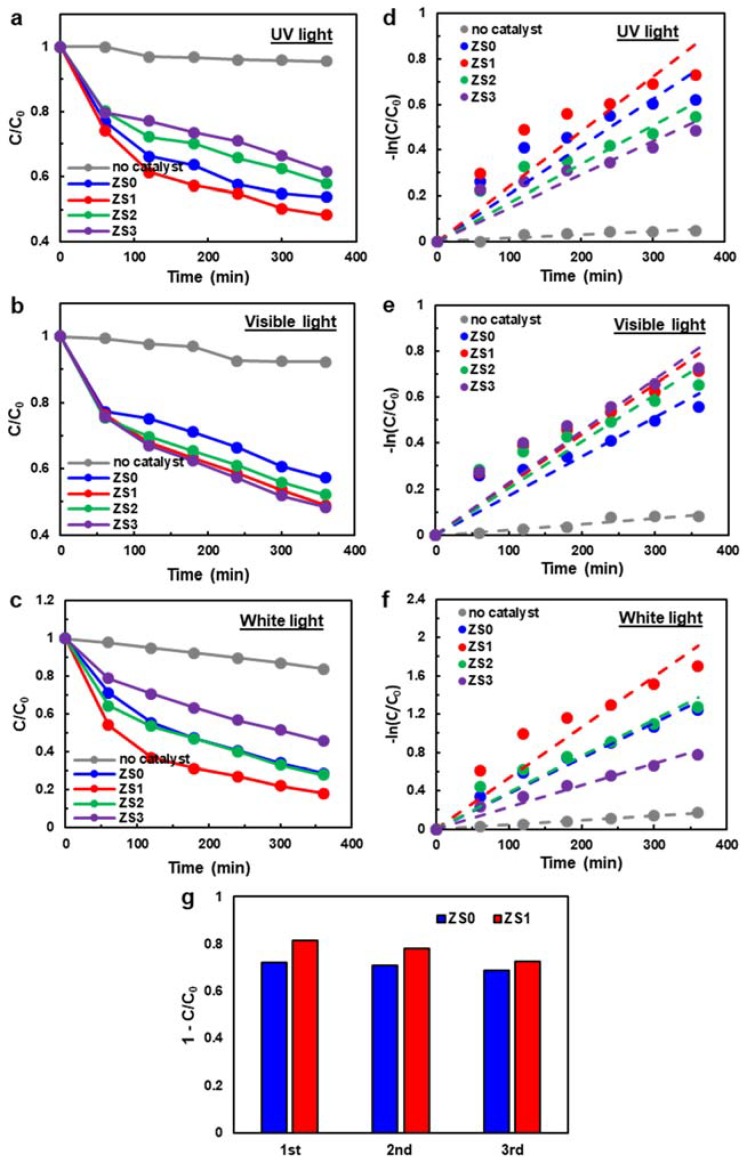
The photodegradation performance of pristine ZnO NWs (ZS0) and three Si-NCs/ZnO-NWs hybrids (ZS1, ZS2, ZS3) under (**a**) UV light, (**b**) visible light and (**c**) white light. (**d**–**f**) The corresponding kinetic plots of pristine ZnO NWs and three Si-NCs/ZnO-NWs hybrids. (**g**) The recyclability test under white light for ZS0 and ZS1.

Figure 6a–c shows the photodegradation performance of pristine ZnO NWs and three Si-NCs/ZnO-NWs hybrids under UV light, visible light and white light. An MB dye solution was used as a model pollutant, and was brought to an absorption–desorption equilibrium after keeping it in the dark for 30 min (see Supplementary Information Figure S3). Note that the degradation testing was performed on the SiNC/ZnO-NW hybrid structures on a Si substrate immersed in the solution. Therefore, the photodegradation performance from this immobilized catalyst platform is inferior to that of the slurry catalyst system [49], and it is not our intention to compare results from these two very different platforms. Rather, the goal is to compare the pristine ZnO-NW sample and hybrid samples under various lighting conditions. The absorption spectra (i.e., peak height) taken from the dye after being irradiated for different time intervals represent the degraded MB concentration. The degradation profile is thus the concentration (*C*) at the degradation time normalized by the initial concentration (*C*_0_). Photolysis of a clean MB dye solution was performed without a catalyst to measure dye photosensitization. This control was carried out under the same degradation conditions and compared to the catalyst-added ones. Less than 8% degradation was observed for UV and visible light conditions when irradiated for 6 h. For white light, 16% of MB was degraded within 6 h.

As shown in Figure 6a, the degraded MB concentration from ZS0 and ZS3 reached the maximum and minimum absorption of 55% and 35%, respectively, in the first 6 h. The pristine ZnO NW sample (ZS0) exhibited a degradation rate similar to ZS1, but higher than ZS2 and ZS3, under UV light. As discussed earlier from the PL data, the thick Si-NC layer atop ZnO NWs may block the incident UV light for ZS2 and ZS3, in which the ZnO NWs in the hybrids absorbed fewer photons and generated fewer charge carriers in comparison to the pure ZnO NWs. The superior performance of ZS1 and ZS2 versus ZS3 suggests that the degradation rate depends on the Si-NC layer thickness. Alternatively, the 365 nm UV light irradiation exceeds the ZnO bandgap (3.37 eV), and so may generate electron–hole pairs more efficiently than Si-NCs. The enhanced degradation rate will also depend on the ZnO surface area exposed to the dye molecules. Thus, ZS3 is less favourable as a catalyst under UV light irradiation. Electron accumulation could occur due to the slow charge carrier transport under UV light irradiation, which will be discussed in the section on the band alignment of the hybrid structures.

The Si NCs with a narrower band gap broaden the photocatalyst response spectrum, while the ZnO NWs alone, with a wide band gap, have a limited response in the UV spectral region. Photocatalytic degradation under visible light (Figure 6b) was used to elucidate the mechanism of the hybrid photocatalysts. Most UV and IR light was filtered out (around 97% blocked), and the intensity of the visible light was reduced by approximately 30%. The trend of the degradation behaviour is opposite to that of the UV light condition (see Figure 6a). The maximum degradation of MB was seen with ZS3, while the least occurred with ZS0. The pristine ZnO sample (ZS0) didn’t absorb the visible light efficiently due to its large band gap, and therefore produced fewer charge carriers and oxidants for photodegradation. The three hybrid structures show little (or minor) difference in degradability. However, the fact that ZS3 shows slightly higher degradability than ZS1 or ZS2 suggests that the role of charge transfer/injection between Si NCs and ZnO NWs is insignificant compared to that of oxidants generated on the Si-NC surface.

The photodegradation performance of pristine ZnO NWs and SiNCs/ZnO-NWs hybrids under white light (IR cut off) (see Figure 6c) differs from those under UV or visible light. The overall degradability follows the sequence of ZS1 > ZS2 ≈ ZS0 > ZS3, respectively. Since white light consists of both the visible and UV spectral regions, the photodegradability under white light exhibits the combined effects from either spectral case. In essence, the higher the Si NC loading of the hybrid structure, the poorer the degradation performance with UV. However, the opposite occurred with visible light. The net effect is seen in Figure 6c, but a detailed explanation requires a deeper understanding of the hybrid structures. Preferential light absorption of ZnO or Si in each spectral region is compounded by the relative placement of the nanostructures (i.e., the incident light is always absorbed by the Si-NC layer first, thus creating the shadowing effect on the ZnO NWs) and the overall absorption efficiency. It is interesting to note that the degradation performance of ZS1 was significantly better (more than 15%) than that of ZS0 or ZS2. The enhanced charge separation and corresponding photogenerated excitons, possibly related to the low ZnO NBE PL intensity of ZS1, may be responsible for the improved behaviour.

The photodegradation performance of the MB dye by the pristine ZnO NWs (ZS0) and Si-NCs/ZnO-NWs hybrids (ZS1, ZS2, ZS3) was further evaluated using a pseudo-first-order kinetic model [15] (i.e., the Langmuir–Hinshelwood model in the limit of a dilute dye concentration): $-\ln\left(C/C_{0}\right)=kt$, where *k* is the apparent reaction rate constant and *t* is the irradiation time. The *k* values can be obtained from the slope of the linear lines that best fit the plots of $-\ln\left(C/C_{0}\right)$ versus *t,* as shown in Figure 6d–f. Table 1 summarizes the obtained *k* values for ZS0 to ZS3 under three different illumination conditions. Again, the comparison of the *k* values between the different illumination scenarios is not appropriate because the illumination intensity is different for each light source. However, when compared under the same illumination, the rate constant is highest for ZS1 in case of UV light, ZS3 for visible light, and ZS1 for white light. It is interesting to note that the datapoints in Figure 6d–f exhibit significant deviations from the linear behavior and that the apparent reaction rates decrease as time progresses. It can be hypothesized that this decrease in *k* may come from the immobilized platform, in which the diffusion of reactants to the catalyst surface becomes a rate-limiting step.

One of the important aspects in developing immobilized photocatalysts is recyclability. If SiNCs/ZnO-NWs are easily dislodged from the glass substrate during the degradation experiments, their recyclability would be significantly hampered. Photodegradation experiments were repeated under white light illumination three times, and their results on ZS0 and ZS1 are shown in Figure 6g. Despite a slight decay in performance over time, no significant performance decline is observed during the recyclability test. In addition, few ZnO NWs were found in the remaining degraded solution when the filtrated paper was inspected under the light microscope.

### 3.5. Band Structures of Si-NCs/ZnO-NWs

In general, photodegradation activity is governed by the amount of participating oxidants, such as superoxide anions (·O_2_^−^) and hydroxyl radicals (·OH), available for degradation reactions. Since these oxidants are produced by the photogenerated charge carriers of the photocatalysts, elucidation of the Si-NCs/ZnO-NWs hybrid band structures is essential. The energy level and band configuration of the Si-NCs/ZnO-NWs hybrid structures can be determined using electronegativity and the measured bandgap energy. The band positions of each semiconductor at the point of zero charge can be calculated by the following equations

*E*_CB_ = *χ* − 0.5 *E*_g_ + *E*_0_ (1)

**Figure 7:**
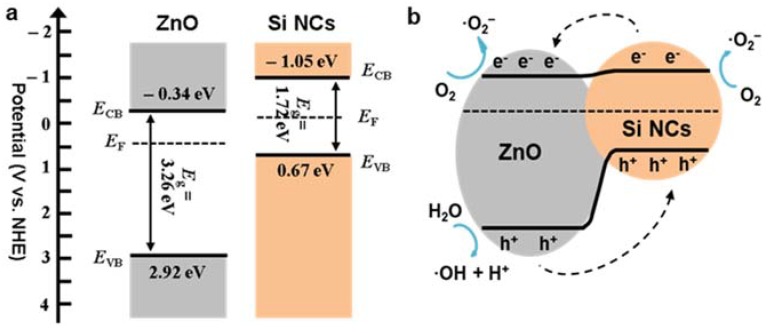
(**a**) The band structures of the Si-NCs and ZnO-NWs, (**b**) the energy diagram of the Si-NCs/ZnO-NWs hybrid.

*E*_VB_ = *E*_g_ + *E*_CB_ (2)

where *E*_CB_ and *E*_VB_ represent the conduction band (CB) and valence band (VB) edge potentials, respectively, *E*_g_ is the band gap energy, *χ* is the absolute electronegativity of the semiconductor, and *E*_0_ is a scaling factor which relates the reference electrode redox level to the vacuum level: *E*_0_ = -4.5 eV for the normal hydrogen electrode (NHE) [50]. *χ* (also knowns as Mulliken electronegativity) is defined as the geometrical mean of the absolute electronegativity of the constituent atoms, i.e., *χ*_Zn_ = 4.45 eV and *χ*_O_ = 7.54 eV, so $\chi_{\mathrm{ZnO}}=\sqrt{\chi_{\mathrm{Zn}}\chi_{O}}=$ 5.79 eV [51]. *χ* for bulk Si is 4.61 eV, but its nanocrystal form may have a different value. *χ* for Si nanocrystal or quantum dot has been rarely reported and should lie between bulk Si (*χ*_Si-bulk_) and a single atom. The absolute electronegative (*χ*) is related to the ionization energy (*I*) and the electron affinity (*A*) by *χ* = (*I* + *A*)/2. Melnikov et al. presented calculations for *A* and *I* for Si-NCs, and both *I* and *A* scale with the radius *R* of the nanocrystal as [52]

(3) $$I^{\;}\left(R\right)=I_{\mathrm{bulk}}+\frac{I_{0}}{{\left(R/a_{B}\right)}^{l_{I}}}$$

(4) $$A^{\;}\left(R\right)=A_{\mathrm{bulk}}-\frac{A_{0}}{{\left(R/a_{B}\right)}^{l_{A}}}$$

where *a_B_* is the Bohr radius (0.529 Å) and the scaling parameters *I_0_*, *A_0_*, *l_I_*, and *l_A_* are given as 44.4, 23.5, 1.2, and 0.9 eV, respectively [52]. *I* and *A* for bulk Si are 4.8 and 4.1 eV, respectively [52]. The average Si-NC in our study is around 5 nm (i.e., *R* = 25 Å), so *I* = 5.23 eV, *A* = 3.37 eV, and *χ*_Si-NC_ = 4.3 eV. The measured bandgaps of ZnO NWs and Si-NCs were 3.26 and 1.72 eV, respectively, and were obtained from photoluminescence and/or diffuse reflectance spectra (see the previous sections). The values of *E*_g_, *E*_CB_ and *E*_VB_ for ZnO NWs and Si-NCs (*E*_CB_ and *E*_VB_ are expressed in terms of NHE) are summarized in Table 2 and plotted in Figure 7a. Figure 7b shows a speculative energy diagram for the ZnO NWs and Si-NCs hybrid structure.

Figure 7b may apply to the near-optimal hybrid structure of ZS1, in which a very thin layer of Si-NCs were coated over the ZnO NWs. Then, we can assume that the recombination of the photogenerated excitons in Si-NCs is insignificant. Upon irradiation, the Si-NCs absorb the photons, releasing photogenerated electrons that either travel to the Si-NC surface to take part in the reduction reaction—converting oxygen molecules in water into superoxide anions—or translate to the hybrid interface and are injected into the ZnO NW conduction band (CB). Meanwhile, the valance band of Si-NCs accepts additional photogenerated holes transferred by ZnO NWs, oxidizing H_2_O to ·OH for further pollutant oxidization. On the other hand, excess photogenerated electrons of ZnO NWs supplied by Si-NCs yield ·O_2_- from O_2_. Therefore, the facilitated charge carriers produce abundant oxidants to degrade the pollutant, resulting in improved photocatalysis. As mentioned above, the barrier caused by charge carrier transport further impedes electron transfer from the Si-NCs. This phenomenon, which emerges with more concentrated Si-NCs, reduces photocatalytic degradation by the Si-NCs, which explains why heavier Si-NCs loading slows degradation [39].

## 4. Conclusions

In this work, we described the synthesis and characterization of Si-NCs/ZnO-NWs hybrid structures as promising sunlight-harnessing photocatalysts. Hydrothermal synthesis and low-pressure non-thermal plasma growth produced highly crystalline ZnO NWs and Si NCs, respectively. The formation of well-defined hybrid structures on the substrates was confirmed through the morphology, composition, and crystallinity of the ZnO NWs and the hybrid nanostructures. The optical properties and functionality of the hybrid structures were investigated as a function of the Si-NC loading. The photodegradation performance of the pristine ZnO-NW and hybrid samples was tested on the immobilized platform under different lighting conditions, including UV, visible, and white light, to investigate the impact of the hybrid structures on photocatalysis. The improved photocatalytic activity of the hybrid with the optimal Si-NC loading reflected the extended light sensitivity of the hybrid structures in the UV to visible, due to more effective charge separation (i.e., suppressed exciton recombination), and increased exciton lifetime. Pristine ZnO-NW and Si-NCs/ZnO-NWs hybrid samples were immobilized on a flat substrate that provided a common platform for comparing photocatalytic performances and optical characteristics. These Si-NCs/ZnO-NWs hybrid structures may have utility beyond photodegradation and may improve the performance of various optical devices.

## Figures and Tables

**Table 1:** Values of the apparent pseudo-first-order rate constant, *k*, obtained from the photodegradation experiments shown in Figure 6a–c.

	*k* (min^−1^)		
	UV	Visible	White
no catalyst	0.0001	0.0003	0.0005
ZS0	0.0021	0.0017	0.0037
ZS1	0.0024	0.0022	0.0053
ZS2	0.0017	0.0020	0.0038
ZS3	0.0015	0.0023	0.0023

**Table 2:** Summary of the values of the conduction band and valence band edge potentials (*E*_CB_ and *E*_VB_) and bandgap (*E*_g_).

	*E*_CB_	*E*_VB_	*E*_g_
ZnO NWs	−0.34 eV	2.92 eV	3.26 eV
Si-NCs	−1.05 eV	0.67 eV	1.72 eV

## Supplementary Materials

The following are available online at https://www.mdpi.com/2079-4991/10/3/491/s1, Figure S1. The top-down and cross-sectional scanning electron microscopy (SEM) images of the thick over-deposited ZnO-NWs/Si-NCs hybrid sample; Figure S2. The top-down SEM views of the four samples and corresponding EDS elemental-mapping spectra for Zn, O, and Si; Figure S3. Degradation result (relative concentration of methylene blue in water) as a function of time when the ZS1 sample is immersed in the solution and under the dark condition. The other samples exhibited similar trends, reaching an absorption–desorption equilibrium within 30 min.
