# Supplementary material for: Si Nanocrystals/ZnO Nanowires Hybrid Structures as Immobilized Photocatalysts for Photodegradation
Source: Nanomaterials (Basel). 2020 Mar 9;10(3):491. doi: 10.3390/nano10030491 (PMC7153658; doi:10.3390/nano10030491)
Supplement: Supplementary file 1 [file nanomaterials-10-00491-s001.pdf]

Supplementary Information

Si Nanocrystals/ZnO Nanowires Hybrid Structures as Immobilized Photocatalysts for Photodegradation

Yaozhong Zhang [1], Rajib Mandal [2], Daniel C. Ratchford [3], Rebecca Anthony [2] and Junghoon Yeom [2,*]

[1] Department of Electrical and Computer Engineering, Michigan State University, East Lansing, MI 48823, USA.; yzzhang@egr.msu.edu

[2] Department of Mechanical Engineering, Michigan State University, East Lansing, MI 48824, USA.; rajib.mandal@intel.com (R.M.); ranthony@msu.edu (R.A.)

[3] Code 6178, Chemistry Division, U.S. Naval Research Laboratory, Washington, DC 20375, USA.; daniel.ratchford@nrl.navy.mil

* Correspondence: jyeom@egr.msu.edu; Tel.: +1-517-432-9132

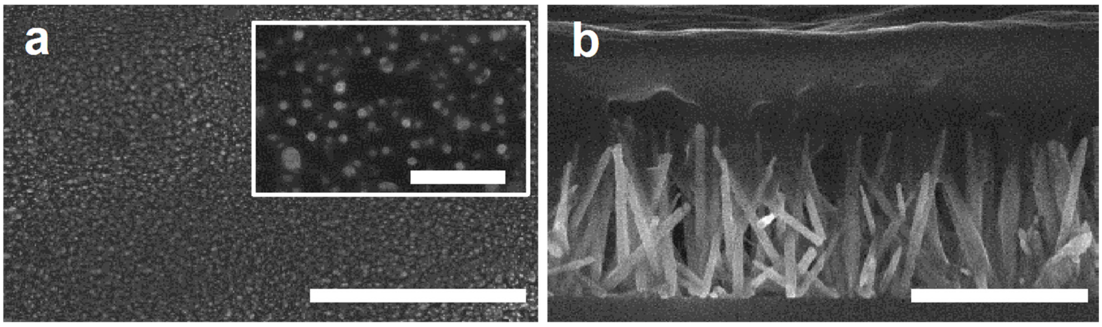

Figure S1. The top-down and cross-sectional scanning electron microscopy (SEM) images of the thick over-deposited ZnO-NWs/Si-NCs hybrid sample. Scale bar: 10 μm in large view and 500 nm in the inset.

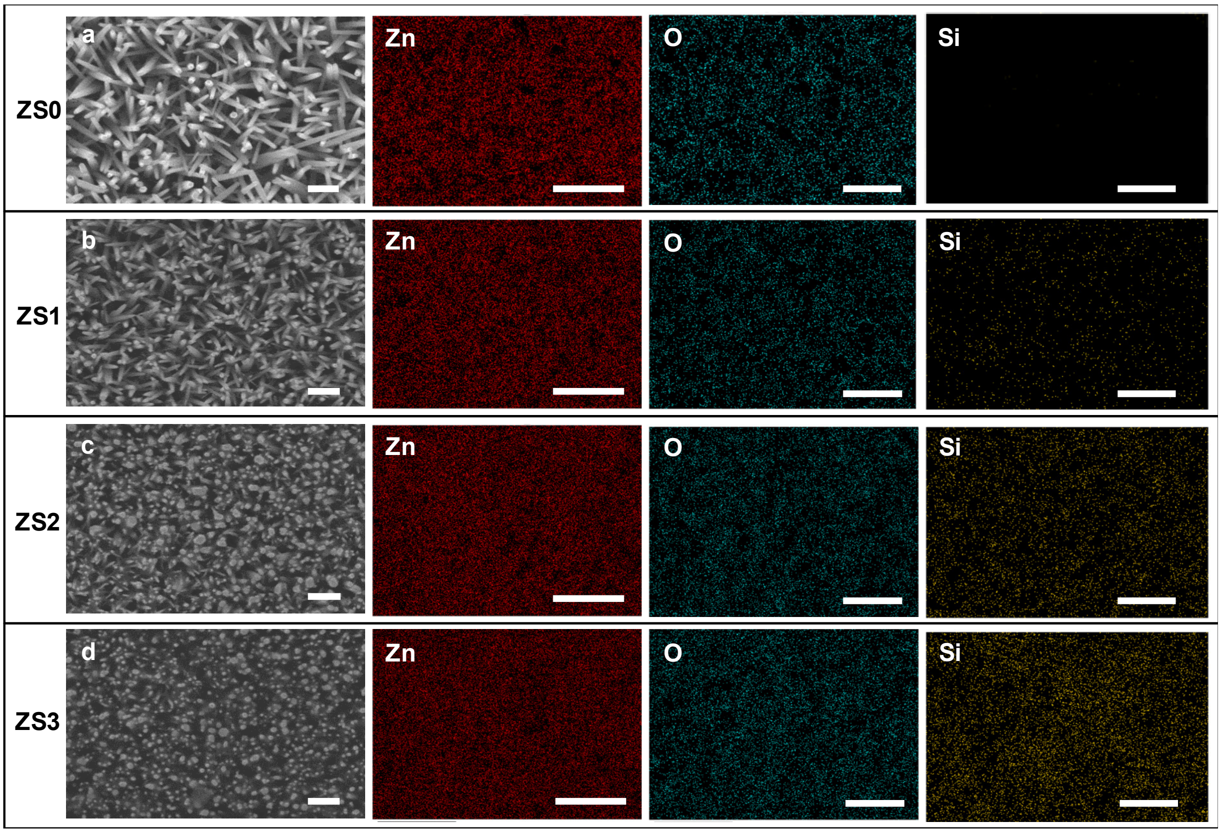

Figure S2. The top-down SEM views of the four samples and corresponding EDS elemental mapping spectra for Zn, O, and Si. Scale bar: 1 µm in large view and 2 µm in EDS mapping images.

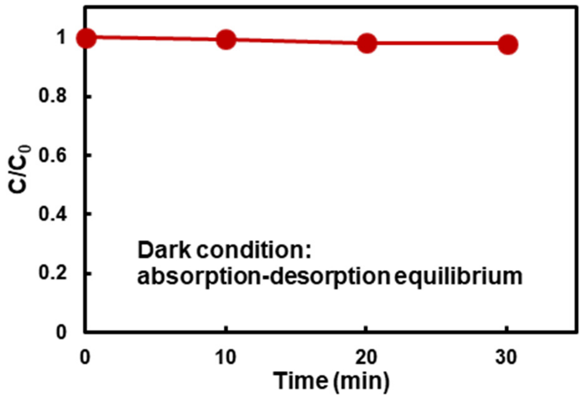

Figure S3. Degradation result (relative concentration of methylene blue in water) as a function of time when the ZS1 sample is immersed in the solution and under the dark condition. The other samples exhibited the similar trends, that is, reaching an absorption-desorption equilibrium within 30 min.
